# Nano-structure fabrication of GaAs using AFM tip-induced local oxidation method: different doping types and plane orientations

**DOI:** 10.1186/1556-276X-6-550

**Published:** 2011-10-06

**Authors:** Jung-Joon Ahn, Kyoung-Sook Moon, Sang-Mo Koo

**Affiliations:** 1 Department of Electronic Materials Engineering, Kwangwoon University, Seoul 139-701, South Korea; 2 Department of Mathematics and Information, Kyungwon University, Seongnam 461-701, South Korea

## Abstract

In this study, we have fabricated nano-scaled oxide structures on GaAs substrates that are doped in different conductivity types of p- and n-types and plane orientations of GaAs(100) and GaAs(711), respectively, using an atomic force microscopy (AFM) tip-induced local oxidation method. The AFM-induced GaAs oxide patterns were obtained by varying applied bias from approximately 5 V to approximately 15 V and the tip loading forces from 60 to 180 nN. During the local oxidation, the humidity and the tip scan speed are fixed to approximately 45% and approximately 6.3 μm/s, respectively. The local oxidation rate is further improved in p-type GaAs compared to n-type GaAs substrates whereas the rate is enhanced in GaAs(100) compared to and GaAs(711), respectively, under the identical conditions. In addition, the oxide formation mechanisms in different doping types and plane orientations were investigated and compared with two-dimensional simulation results.

## Introduction

Atomic force microscopy (AFM) is considered as a promising tool to analyze and modify the nano-scaled structures and devices, and thus AFM-based local oxidation (AFM-LO) process has been intensively investigated to fabricate and modulate nano-structures and devices such as field-effect transistors and single-electron transistors with various samples including metals, semiconductors, and even insulators [1,2]. The AFM-LO process is basically an anodic oxidation, where the AFM tip and substrate act as the cathode and anode, respectively. Thus, by applying a negative bias to a conductive AFM tip, an intense localized electric field is created at the substrate close to the tip and the mechanism of AFM-LO has been understood in terms of field-induced oxidation, which requires larger local electric field than the critical electric field of typical about 1 V/nm to dissolve the water molecules to H+ and OH- ions in water bridge formed around the tip [3,4] and the sample surface. Then, OH- ions are transported to the positively biased sample surface in the direction of the electric field and form the oxide structures as reacting with atoms in the sample surface [3-6].

Recently, AFM-LO has been investigated primarily on Si [5-8] and further extended to wide band gap semiconductors [9], graphene [10], and other compound semiconductors such as GaAs and AlGaAs [11-16]. In case of GaAs, AFM-LO on heavily doped p-type GaAs has been studied to improve aspect ratios and lateral resolutions of oxide structures [16]. However, the AFM local oxidation studies comparing different doping types and plane orientations of GaAs have not been reported.

In this study, we systematically performed AFM-based local oxidation on both n- and p-GaAs of different plane orientations with (100) and (711), respectively. We used a contact mode AFM for oxidation [17], which allows varying the loading forces of the tip onto the sample surfaces as the oxide structure is formed. The influence of the applied voltages on the formation of local oxide was also investigated and compared with numerical simulations [18,19].

## Experimental

A commercial AFM (N8 ARGOS, Bruker AXS Inc., Madison, WI, USA) was used to perform AFM-LO in contact mode AFM and topography measurement in non-contact mode AFM. A Si cantilever with a Pt-coated conductive tip (ANSCM series, Appl Nano, Santa Clara, CA, USA) having a diameter of approximately 100 nm was used. The spring constant and the resonance frequency were set to 3 N/m and 70 kHz, respectively. Before performing AFM-LO, the GaAs samples were cleaned by NH_4_OH/H_2_O mixtures to remove metal contaminations and native oxides. For environmental control, the microscope was placed into a closed box with the relative humidity around 45%.The local oxide patterns were generated on n- and p-type GaAs(100) and GaAs(711), respectively, with a doping concentration of approximately 10^19 ^cm^-3^, at room temperature during the experiments. The oxide structures were formed electrochemically on the GaAs reactive surface by applying a negative bias voltage between the sample surface and the AFM probe. The electrical field was then created between the native oxide layer and the substrate, which caused the oxyanions (OH-) to drift through the oxide film [3-6]. During the AFM local oxidation in contact mode, the tip applied bias was varied in the range of 5 to 15 V and the tip loading force was modulated from approximately 60 nN to approximately 180 nN. The scan speed was fixed to 6.028 μm/s, during the process.

During the AFM local oxidation in contact mode, the voltage was varied in the range of 5 to 15 V and the tip loading force was modulated from approximately 60 nN to approximately 180 nN. In addition, the chemical composition of the grown local oxides was analyzed by an Auger electron spectroscopy (AES) system with a Schottky field emission electron source. Numerical simulations were performed by using COMSOL Multiphysics software (FEMLAB, Burlington, MA, USA).

## Results and discussion

**Figure 1:**
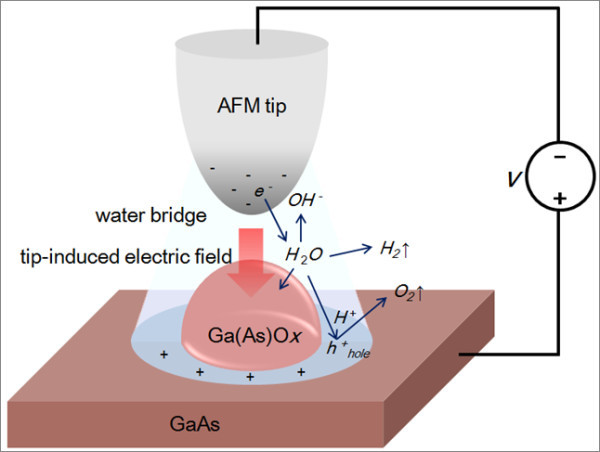
**Schematics of the chemical reactions and species involved in the AFM local oxidation process**. Induced by applying bias voltage on AFM tip in air.

The mechanism of local oxidation on the GaAs surface by contact mode AFM using Pt-coated probe is described in Figure 1. As an AFM probe is approaching to a GaAs, a water bridge is developed around the tip-sample junction due to the capillary force. The AFM probe performs as an electrode at the sample surface which is anodically biased, while the layer of absorbed water on the surface dissociates by a high electric field and acts as an electrolyte producing this electrochemical reaction. The chemical reactions and charge transfer processes can be considered as follows [12]:

1. Reactions at the GaAs surface:

$$\begin{array}{c} \text{2GaAs}+\text{6}{\text{H}}_{\text{2}}\text{O}+{\text{12h}}_{\text{hole}}^{+}\to \text{G}{\text{a}}_{\text{2}}{\text{O}}_{\text{3}}+\text{A}{\text{s}}_{\text{2}}{\text{O}}_{\text{3}}+\text{12}{\text{H}}^{+}\\ \text{6}{\text{H}}_{\text{2}}\text{O}+{\text{12h}}_{\text{hole}}^{+}\to \text{3}{\text{O}}_{\text{2}}↑+\text{12}{\text{H}}^{+} \end{array}$$

2. Reaction at an AFM probe:

$$\text{12}{\text{H}}_{\text{2}}\text{O}+\text{12}{\text{e}}^{-}\to \text{6}{\text{H}}_{\text{2}}↑+\text{12O}{\text{H}}^{-}$$

3. Reaction in water:

$$\text{12}{\text{H}}^{+}+\text{12O}{\text{H}}^{-}\to \text{6}{\text{H}}_{\text{2}}\text{O}$$

Here, h^+^_hole _represents positively charged holes on the GaAs surface. During the oxidation process, it is expected that the H+ and OH- ions generated at the GaAs surface and an AFM probe will recombine immediately according to the recombination reaction in water and Ga_2_O_3 _and As_2_O_3 _are formed on the reactive surface as Ga(As)O*x *is formed.

The oxidation kinetics reported for Si [5-8] and GaAs [12-14] indicate that regardless of the materials, the observed self-limiting growth behavior is universal in AFM tip-induced oxidation and its kinetics shows some differences with the Cabrera-Mott theory [20] for field-induced oxidation. In 1997, Avouris et al. [8] proposed that the growth kinetics can be described as d*h*/d*t *∝ exp(-*h*/*l*_c_), where *h *is the oxide thickness at time *t *and *l*_c _is a characteristic decay length depending on the anodization voltage. This implies that lower scan rate can be more effective in fabricating oxide structures. Other than the scan rate and anodization voltage, in performing AFM local oxidation with contact mode AFM, we need to consider the tip loading force. The height and aspect ratio of oxide structures can be improved with a proper loading force integrated with the tip-surface electric field.

**Figure 2:**
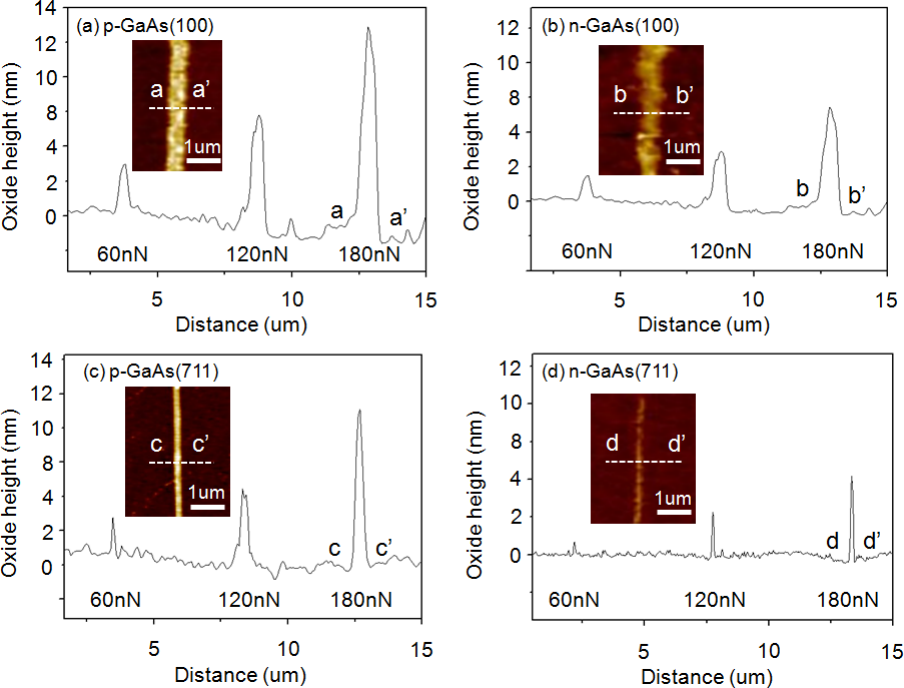
**An AFM images displaying the oxide lines**. Formed at (**a**) p-type GaAs(100), (**b**) n-type GaAs(100), (**c**) p-type GaAs(711), and (**d**) n-type GaAs(711) with varying loading forces of 60, 120, and 180 nN and applying tip voltage of 5 V.

Figure 2 depicts a cross section of AFM local oxide line patterns formed on p-GaAs(100), n-GaAs(100), p-GaAs(711), and n-GaAs(711) substrates, respectively. The patterns in Figure 2 were obtained by using a constant negative tip voltage of 5 V at the different oxidation loading forces of 60, 120, and 180 nN.

By varying the loading forces from 60 to 180 nN with a fixed applied negative bias of 5 V, the height of modified oxide structures was controlled in the range of approximately 3 nm to approximately 14 nm. As the loading force increases from 60 to 180 nN, the height of the oxidation pattern structures increases.

It is interesting to note that the oxide structures that are formed in p-GaAs(100) is about doubled in height to that of n-GaAs(100). We observed that increasing loading force can result in larger and higher oxide patterns on GaAs with each doping type. It has been reported that increasing applied voltages can enhance the electric field between AFM tip and sample surface and cause larger oxide formation [5-7].

**Figure 3:**
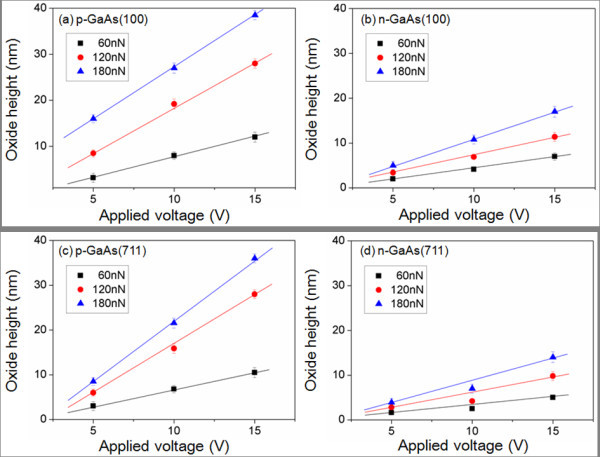
**AFM local oxidation results**. Of (**a**) p-GaAs(100), (**b**) n-GaAs(100), (**c**) p-GaAs(711), and (**d**) n-GaAs(711) as a function of the applied bias voltages and the loading forces.

Figure 3 represents the height of oxide patterns generated on GaAs substrates with different doping types and plane orientations, as a function of applied voltages from 5 to 15 V. During the local oxidation, tip loading forces in the range of 60 to180 nN were induced. The oxide patterns are formed at loading force of over 60 nN with an applied voltage of 5 V, which is a threshold bias voltage considering the circumstances.

The oxide heights of p-type GaAs(100) are varied from approximately 3.2 nm to approximately 39 nm which is clearly higher than that of n-GaAs(100). In the case of a n-GaAs(711), the oxide is rarely formed to be around 1.6 to 2.8 nm. It is observed that the oxide height increases, as the anodization voltage and as the loading force is increased, as can also be seen from the linear fit to experimental data. In order to control the size of oxide patterns, the anodization voltages should also be modulated in close relation to the tip loading forces.

In case of p-GaAs(100), the slope extracted from the linear fit varies from 1.44 to 2.7, whereas the slope for n-GaAs(100) increases from 0.28 to 1.03, which indicates that the oxidation rate p-type GaAs is not only high for but is also more sensitive to the bias change than for n-type GaAs.

In order to investigate the impact of applied voltages and loading forces on tip-induced electric field, we performed two-dimensional simulations (COMSOL Multiphysics software, FEMLAB).

**Figure 4:**
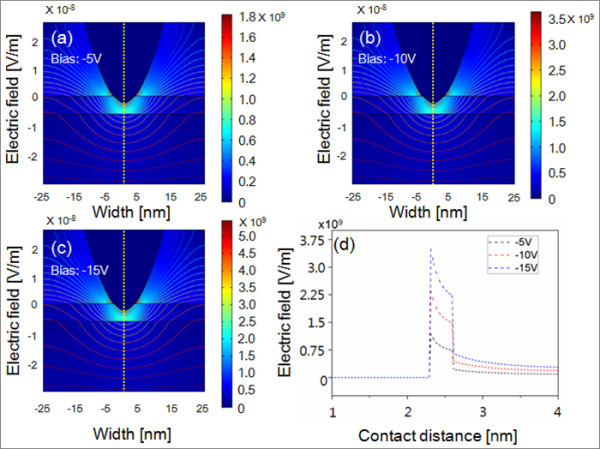
**Contoured image of electric field between AFM probe and GaAs surface at different applied voltages**. (**a**) -5 V, (**b**) -10 V, (**c**) -15 V, and (**d**) the electric field profile along the vertical cross-sectional lines for different bias conditions.

**Figure 5:**
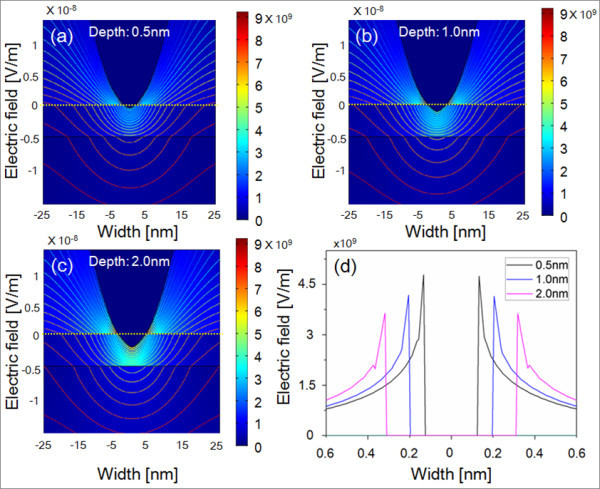
**Contoured image of electric field between AFM probe and GaAs surface at different penetration depth**. (**a**) 0.5 nm, (**b**) 1.0 nm, (**c**) 2.0 nm, and (**d**) the electric field profile along the horizontal cross-sectional lines for different depth conditions.

By combining the definition of potential with Gauss' law and the equation of continuity, it is possible to derive the following Poisson's equation:

$$-\nabla \cdot \left(\varepsilon_{0}\varepsilon_{\text{r}}\nabla \text{V}\right)=\rho$$

where *ε*_0 _is the permittivity of free space, *ε*_r _is the relative permittivity, and *ρ *is the space charge density. The basic geometries are shown in Figures 4 and 5, and the regions are coupled via boundary conditions; **n**(*D*1- *D*2) = 0 on the surfaces of substrate as continuity condition and **n**·*D *= 0 on all outer boundaries as symmetry condition and *V *= *V*_0 _electric potential boundary condition, where **n **is the outer normal vector to the boundary.

As shown in the electric field and potential distributions of Figure 4, an intense localized electric field maximum is created at the edge of the tip close to the substrate for different bias conditions of -5, -10, and -15 V. The electric field is enhanced around the edge of AFM tip and substrate region. Figure 4d compares the electric field profile along the vertical cross-sectional lines for different bias conditions. As observed in the experiments, the increased bias results in an increase in a local maximum electric field and thus improved local oxidation.

**Figure 6:**
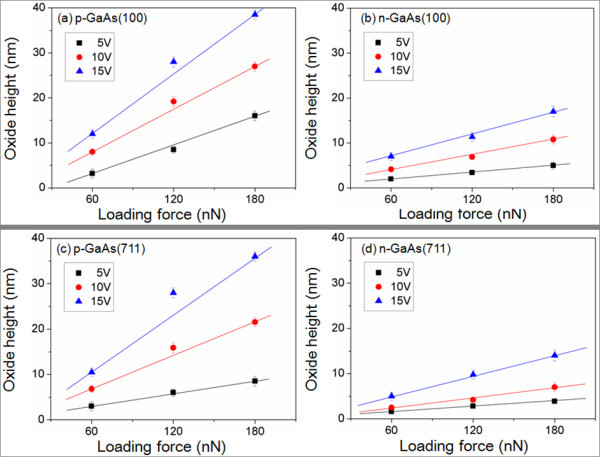
**AFM local oxidation results**. Of (**a**) p-GaAs(100), (**b**) n-GaAs(100), (**c**) p-GaAs(711), and (**d**) n-GaAs(711) as a function of the applied bias voltages and the loading forces.

Figure 6 shows the loading force-dependent local oxide height for GaAs with different doping types and plane orientations. The loading forces are changed from 60 to 180 nN. It can be seen that the oxide height almost linearly increases as when the loading force is increased. The slope, from the oxide height versus loading force plots of Figure 6, varies from 0.96 to 2.3 for p-GaAs, whereas the slope changes from 0.48 to 1.3 n-GaAs, depending on the applied bias. This behavior is similar to the experimental results on bias dependence shown in Figure 3. It is thus crucial to modulate the distance between AFM tip and oxide-substrate surface so as to control the oxidation rate.

Figure 5 shows the electric field distributions and equi-potential lines in the AFM tip and substrates structures with different tip-penetration depths of 0.5, 1.0, and 2.0 nm, respectively. As shown in Figure 5d, the maximum electric field forms around the edge of the tip and the surface, and therefore the distance between the maximum fields increases as the penetration depth increases. Note that the level of maximum electric field does not change much and still well above threshold electric field of approximately 10^9 ^V/m. The penetration depth, which is basically deformation of the formed oxide or substrate through water layer, is dependent on the applied loading force to the tip, which suggests improved oxidation for a higher loading force.

**Figure 7:**
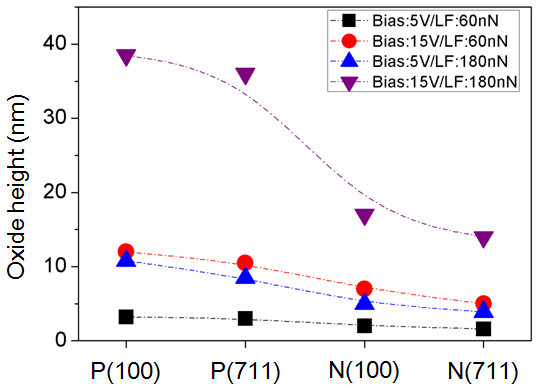
**Oxide height profiles of p-GaAs(100), n-GaAs(100), p-GaAs(711), and n-GaAs(711)**. As a function of the applied bias voltages and the loading forces.

Figure 7 summarizes the height of oxide patterns for the GaAs samples with different doping types and plane orientations, as a function of applied voltages (5 and 15 V) and loading forces (60 and 180 nN). It can be observed that the oxide height is further improved by adjusting the loading force, for the same applied bias. Comparing the oxide height of different doping type and plane orientation, it is clearly shown that p-GaAs have higher oxidation rate in both plane orientations of (100) and (711). On the other hand, GaAs(100) shows higher oxidation rate than GaAs(711) under the identical conditions.

**Figure 8:**
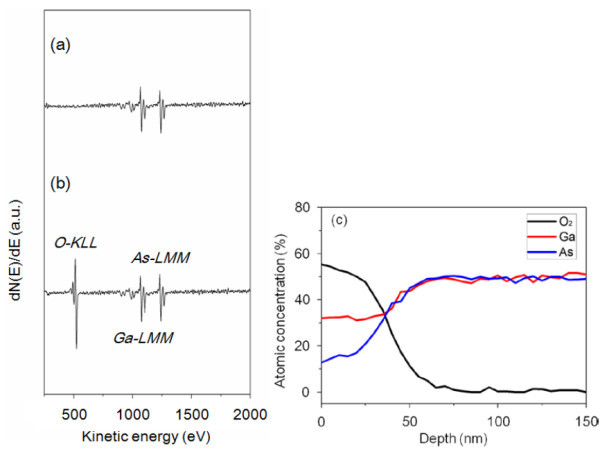
AES profiles of (a) the former oxidized, (b) the oxide patterned intrinsic GaAs(100) surface and (c) the atomic concentration ratio of Ga, As, and O atoms in intrinsic GaAs.

In order to understand the behavior further and to investigate the chemical composition of the oxide structures, AES analysis was conducted on an oxidized area of 5 × 5 μm^2 ^(35 nm to approximately 42 nm oxide height). The Auger spectra taken from the GaAs surface without any local oxidation are compared with the local oxide patterned GaAs as shown in Figure 8a. Both spectra have emission peaks of Ga-*LMM *at approximately 1, 065 eV and As-*LMM *at approximately 1, 225 eV. The emission peak of O*-KLL *Auger electrons having a kinetic energy of approximately 512 eV was detected in patterned area. The atomic concentration at Ga(As)O*x *and GaAs is shown in Figure 8c. The composition ratio of Ga(As)O*x *was as a function of depth by sputtering into the oxidized area about 150 nm. Note that the relative atomic concentration ratio of Ga_2_O_3 _is about two times larger than that of As_2_O_3 _The results suggest that the predominant oxide is Ga_2_O_3_, and therefore improved oxidation on (100) plane orientation has been explained by the different atomic density and surface states between Ga-rich GaAs(100) and As-rich GaAs(711) faces.

## Conclusions

To summarize, the AFM tip-induced local oxidation technique has been used to investigate the oxidized nano-structures on GaAs of different doping types and plane orientations. The local oxide growth rate on GaAs is found to be proportional to both applied voltages and loading forces. Two-dimensional simulation was carried out to investigate the impact of applied voltages and loading forces on tip-induced electric field between AFM tip and GaAs surface.

The experimental results indicate that AFM local oxidation on p-GaAs is further enhanced, compared to n-GaAs, and this can be attributed to the predominant oxide proportion in Ga(As)O*x *that is composed of Ga_2_O_3 _and As_2_O_3_. The atomic concentration in Ga(As)O*x *was analyzed by AES analysis, and the results indicate that Ga(As)O*x *contains both Ga_2_O_3 _and As_2_O_3 _and the atomic concentration of Ga is approximately two times larger than that of As. It supports that the predominant oxide is Ga_2_O_3_. In addition, the AFM local oxidation on different plane orientations, GaAs(100) and GaAs(711), was investigated. The improved oxidation on (100) plane orientation has been explained by the different atomic density and surface states between Ga-rich GaAs(100) and As-rich GaAs(711) faces.

## Competing interests

The authors declare that they have no competing interests.

## Authors' contributions

JJA carried out the AFM local oxidation process and prepared the manuscript initially. KSM participated in data analysis and performed two-dimensional numerical simulations. SMK conceived the study and participated in its design and coordination. All authors read and approved the final manuscript.
